# Uncemented total knee arthroplasty is associated with higher complication rates: a propensity-matched retrospective study

**DOI:** 10.1007/s00402-025-06187-y

**Published:** 2026-02-04

**Authors:** Winston E. Tawiah, Joshua T. Ou, Samuel S. Gay, Jared Wainwright, Adam Nguyen, Joseph C. Wenke, Bardia Barimani

**Affiliations:** 1 The University of Texas Medical Branch at Galveston, Galveston, USA; 2 Department of Orthopaedic Surgery and Rehabilitation, The University of Texas Medical Branch, Galveston, USA; 3 Shriners Hospitals for Children—Galveston, Galveston, USA; 4 Division of Orthopedic Surgery, McGill University, Montreal, Canada

**Keywords:** Total knee arthroplasty, Cemented fixation, Uncemented fixation, Prosthetic joint infection, Orthopedic outcomes, Retrospective study

## Abstract

**Introduction:**

Modern uncemented total knee arthroplasty (TKA), is considered an alternative to the traditional cemented fixation, especially for younger and highly active patients; however, questions remain regarding its comparative complication profile, specifically in the early postoperative period. The purpose of this study was to determine the rate of postoperative complications associated with both cemented and uncemented TKA by using a large, national database.

**Materials and methods:**

We conducted a retrospective cohort study using the TriNetX Research Network, which aggregates deidentified electronic health record (EHR) data from over 127 million patients. Adults who underwent their first primary TKA between October 1, 2012, and October 1, 2020, with a minimum of three years follow-up were included in the analysis. After applying inclusion/exclusion criteria, 4135 cemented TKAs were 1:1 matched to 4135 uncemented TKAs using a propensity score matching based on demographic variables and comorbidity. Follow up assessments were made at 90 day, 1 year, and 3 year intervals included prosthetic joint infection (PJI), revision procedures, aseptic loosening, periprosthetic fracture, dislocation, and all-cause complications, defined as the sum of the prosthetic joint infection (PJI), revision procedures, aseptic loosening, periprosthetic fracture, dislocation.

**Results:**

In the matched sample (*n* = 4,135 per group), cemented TKA was associated with lower rates of PJI at 90 days (OR 0.525, 95% CI 0.334–0.824) and 1 year (OR 0.695, 95% CI 0.505–0.956). Cemented fixation was also associated with reduced all-cause complications at 90 days (OR 0.568, 95% CI 0.361–0.824), 1 year (OR 0.671, 95% CI 0.511–0.882), and 3 years (OR 0.765, 95% CI 0.697–0.963). No clinically significant differences existed between the two groups regarding aseptic loosening or periprosthetic fractures.

**Conclusions:**

Using a large, propensity matched group of patients, we found that Cemented TKA demonstrated statically significant lower rates of PJI and all-cause complications compared with Uncemented TKA. These findings suggest potential differences in short-term outcomes between fixation types, although causality cannot be inferred due to the observational nature of the study and limitations inherent to database research, including coding variability and the absence of implant-level detail. Future studies should include design aspects of the implants, as well as surgeon-specific characteristics and extended follow up.

## Introduction

TKA (total knee arthroplasty), is considered one of the most common orthopedic surgeries and its annual volume continues to rise, particularly among younger and more active patients [1]. It is estimated that approximately 1.3 million TKA surgeries took place in the U.S. in 2020 [2] and projections suggest an increase to approximately 3.5 million procedures annually by 2030 [2]. As the demand for TKA continues to grow, so too does the desire to improve prosthesis longevity and durability, especially for younger patients who typically place greater mechanical stress on their prostheses.

Cemented fixation, which has traditionally been used to stabilize the components in TKA, uses a fast-acting bone cement to bond the prosthetic component to the patient’s host bone [3] and has been successful in providing predictable results. Despite the long history of success for cemented fixation in TKA, cemented fixation is not without limitations, particularly in younger or obese patients. Younger and obese patients are generally at a higher risk for micromotion of the implant, cement fatigue, and aseptic loosening of the implant as compared to other groups of patients [4, 5]. To address these concerns, uncemented fixation techniques were developed with the goal of improving long-term biological fixation and reducing cement-related failure modes by promoting osseointegration [6].

Early generations of uncemented implants, however, faced considerable challenges, including unstable fixation, high rates of aseptic loosening, and increased early revision rates [7, 8]. These shortcomings limited their widespread adoption. With the introduction of new, modern uncemented TKA designs that feature more anatomic geometries and better surface technologies, along with more porous coating, interest in uncemented fixation methods has been renewed. The new designs have shown more stable fixation, less micromotion, and more reliable bone ingrowth, thereby making them more appealing options for younger, more active, or obese patients [9, 10].

Despite the increasing popularity of uncemented TKA, the literature regarding the topic of uncemented TKA remains inconsistent. Some studies have suggested potential benefits of uncemented fixation including shorter operative times and longer term fixation stability. Many studies have also failed to find any statistically significant differences in functional outcomes or complication rates when compared to cemented fixation [11, 12]. For example, a meta-analysis conducted by Parsad et al., which included randomized controlled trials from 2018, indicated that there were no statistically significant differences between the two fixation methods in terms of revision surgery, post-operative infections, or post-operative function [11]. However, the individual studies contained within that analysis were small and likely under-powered to detect statistically significant differences in low frequency complications such as PJI (prosthetic joint infection) or early revision [13].

More recent studies utilizing large administrative databases have suggested that uncemented fixation is associated with a higher incidence of early complications. Forlenza et al. found that uncemented TKA patients had a higher risk of reoperation at one year and developing aseptic loosening and undergoing reoperation at two years as compared to cemented TKA patients [12]. While that study followed patients for up to two years, it did not assess complication rates beyond the early postoperative period. At present, there is a lack of large-scale, propensity-matched studies comparing the outcomes of cemented and uncemented TKA patients beyond 2 years.

Therefore, given the current limitations of existing research in this area, the purpose of this study was to examine complication rates of cemented and uncemented TKA patients utilizing a large national database with a minimum of three years of follow-up. Our aim was to evaluate early and mid-term postoperative complications of both fixation types at multiple time intervals to provide broader context to the evolving discussion regarding fixation choice in TKA.

## Materials and methods

### Records selection

This retrospective cohort study used data from the TriNetX Research Network (TriNetX, Cambridge, MA, USA). The network includes the aggregated electronic health records of over 92 participating healthcare systems and has the capacity to include demographic data and diagnostic codes, both for diagnosis (International Classification of Diseases [ICD]) and procedures (ICD and Current Procedural Terminology [CPT]), as well as prescription medications by their Anatomic Therapeutic Chemical (ATC) classification. Because TriNetX provides only deidentified, aggregated data in the form of counts and statistical summaries, institutional review board approval was not required for this study.

**Fig. 1:**
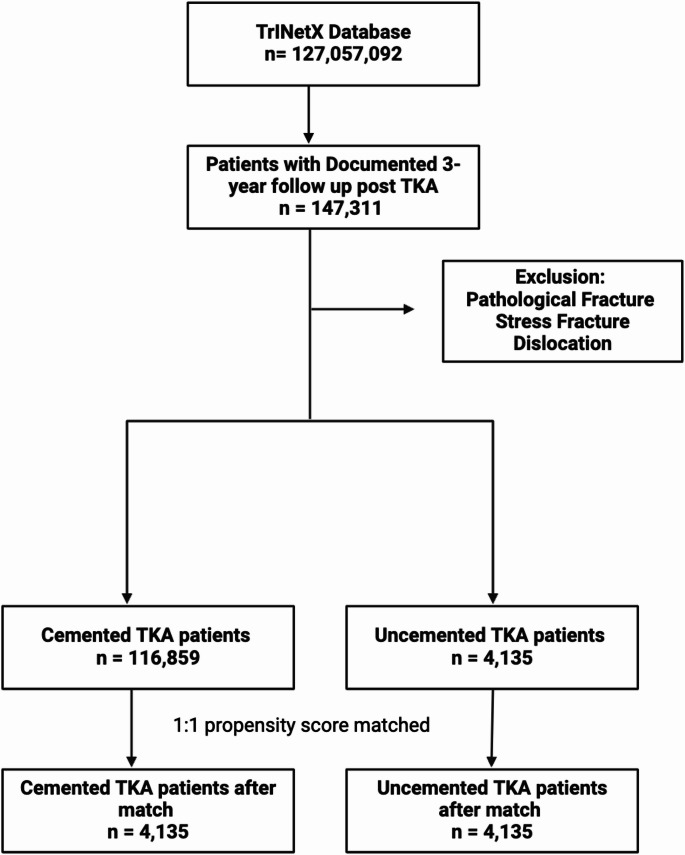
TriNetX selection criteria for TKA patient selection

The database contains records from more than 127 million individuals. Of which, there are 147,311 patients that have a minimum of 3 years of documented post-operative follow-up after having undergone a first-time primary total knee arthroplasty (TKA) between October 1, 2012 and October 1, 2020 (Fig. 1). The timeframe for the study was chosen to provide consistency in the types of modern TKA prosthetics being implanted into the patients, and to assure that there would be sufficient length of time to observe complications, such as aseptic loosening and early revision surgery, that may not develop until later than a short-term follow up timeframe. The inclusion of the length of time for patient follow up (≥ 3 years) allows for the evaluation of both early and intermediate outcomes while maintaining sample size and the diversity of institutions involved. Requiring 5-year follow-up would have substantially reduced the cohort and potentially biased the sample toward centers with longer follow-up. Excluded from the study were those patients that had pathologic fractures, traumatic fractures, revisions of previous TKAs, and tumor-related surgeries. Both cemented and uncemented TKAs performed with or without robotic assistance were included, as TriNetX does not reliably distinguish robotic from manual techniques.

After applying inclusion and exclusion criteria, 116,859 patients who underwent cemented TKA and 4,135 who underwent uncemented TKA were identified. A propensity score matching algorithm that uses greedy nearest neighbor matching without replacement (1:1 ratio) was used to generate comparable groups of patients. Those variables used in the propensity score matching algorithm were selected for inclusion in the algorithm based upon clinical relevance and evidence provided in prior literature; and included obesity/overweight, diseases of arteries, arterioles, capillaries, nicotine dependence, chronic kidney disease (CKD), diabetes mellitus, liver disease, osteoporosis without current pathological fracture, and essential hypertension. The variables were identified utilizing ICD codes. Standardized mean differences (SMDs) were calculated to assess balance between the two cohorts following propensity score matching; SMDs < 0.1 were determined to be acceptable.

## Outcomes

Postoperative outcomes were identified using ICD-10 and CPT codes recorded in the electronic health record at predefined intervals of 90 days, 1 year, and 3 years. Outcomes included dislocation, prosthetic joint infection (PJI), aseptic loosening, periprosthetic fracture, revision procedures, and all-cause complications. Revision was defined broadly to include any subsequent operative procedure involving exchange, removal, or replacement of one or more prosthetic components. Incision and drainage procedures without component exchange or removal were not classified as revisions.

All-cause complications were calculated as the cumulative number of distinct postoperative complications captured by the outcome codes outlined in Appendix 1. These included both medical and surgical events and served as a composite measure of overall postoperative morbidity. Because database-derived outcomes depend on accurate procedural and diagnostic coding, the potential for miscoding or underreporting is acknowledged as an inherent limitation.

### Statistical analysis

Statistical analyses were performed using the TriNetX Live analytics platform. Continuous variables were summarized as means with standard deviations, and categorical variables were summarized as counts and percentages. Group comparisons were performed using odds ratios (ORs) with 95% confidence intervals (CIs). TriNetX estimates odds ratios and 95% confidence intervals using logistic regression models, while Pearson’s Chi-squared tests are used for comparisons of categorical variables. These methods are built into the TriNetX analytics engine and were applied consistently across all outcome comparisons. A p-value < 0.05 was considered statistically significant, and ORs were considered significant when their 95% CIs excluded 1.00.

In order to account for the evaluation of various outcomes at three different time points (90 days, 1-year, and 3-years) that could result in multiple outcomes being compared, Bonferroni adjustment was made to correct for the family-wise error rate. Adjusted alpha values were calculated by dividing 0.05 by the number of comparisons for each outcome category. The adjusted alpha values are indicated in the corresponding tables and figures.

To account for multiple comparisons across time intervals and complication categories to compare statistically, Bonferroni corrections were used when applicable and mentioned on the corresponding figures. In accordance with TriNetX privacy protocols, cells with less than 10 patients have been masked and are represented as 10 patients. The masking was factored into the analyses when it occurred; thus, some of the rarer complication rates will likely be very slightly overestimated. This limitation primarily affects interpretation of uncommon outcomes such as dislocation, early aseptic loosening, and periprosthetic fracture.

## Results

Before propensity score matching, there were statistically significant differences between the cemented and uncemented TKA cohorts across nearly all baseline characteristics, with the exception of nicotine dependence (*p* = 0.290). Patients who underwent uncemented TKA were younger (66.7 ± 9.7 years vs. 73.2 ± 9.5 years for cemented TKA) and generally healthier, as reflected by lower rates of essential hypertension (37.6% vs. 46.0%), overweight/obesity (19.4% vs. 22.6%), diabetes mellitus (14.4% vs. 17.3%), chronic kidney disease (4.16% vs. 6.57%), and other comorbidities (Table 1). These baseline differences demonstrated clinically meaningful imbalance and supported the need for matching.

After 1:1 propensity score matching, all measured covariates showed improved balance between the two cohorts (Table 2). Standardized mean differences (SMDs) for all matching variables were < 0.10, indicating acceptable balance. The matched cohorts each contained 4,135 patients.

**Table 1:** Cohort characteristics comparison between cemented and uncemented TKA patients before matching

Characteristic	Cemented TKA (*n* = 116,859)	Uncemented TKA (*n* = 4,135)	Std. Diff	*P*-value
Age at Index, mean ± SD	66.7 ± 9.69	63.8 ± 9.82	0.2965	< 0.0001
Female	68,855 (59%)	1,909 (46%)	0.2575	< 0.0001
Male	42,859 (37%)	1,824 (44%)	0.1520	< 0.0001
Not Hispanic or Latino	85,026 (73%)	2,693 (65%)	0.1655	< 0.0001
Hispanic or Latino	4,561 (4%)	182 (4%)	0.0250	0.1046
Overweight & Obesity	26,448 (23%)	802 (19%)	0.0795	< 0.0001
Diseases of arteries/arterioles/capillaries	10,037 (9%)	225 (5%)	0.1235	< 0.0001
Nicotine dependence	7,225 (6%)	239 (6%)	0.0170	0.2901
Chronic kidney disease	7,681 (7%)	172 (4%)	0.1072	< 0.0001

**Table 2:** Cohort characteristics comparison between cemented and uncemented TKA patients after matching

Characteristic	Cemented TKA (*n* = 116,859)	Uncemented TKA (*n* = 4,135)	Std. Diff	*P*-value
Age at Index, mean ± SD	63.8 ± 9.84	63.8 ± 9.82	0.0021	0.928
Female	1,918 (46.4%)	1,909 (46.2%)		0.579
Male	1,831 (44.3%)	1,824 (44.1%)		0.843
Overweight & Obesity	780 (18.9%)	802 (19.4%)		0.539
Diseases of arteries/arterioles/capillaries	207 (5.00%)	225 (5.44%)		0.374
Nicotine dependence	203 (4.91%)	239 (5.78%)		0.078
Chronic kidney disease	157 (3.80%)	172 (4.16%)		0.399
Diabetes mellitus	585 (14.1%)	595 (14.4%)		0.753
Liver disease	141 (3.41%)	147 (3.56%)		0.719
Osteoporosis	121 (2.93%)	127 (3.07%)		0.719
Essential hypertension	1,560 (37.7%)	1,555 (37.6%)		0.910

Prior to matching, cemented TKA was associated with significantly lower odds of prosthetic joint infection (PJI) at 90 days (OR 0.714, 95% CI 0.537–0.948), 1 year (OR 0.644, 95% CI 0.523–0.793), and 3 years (OR 0.732, 95% CI 0.605–0.886). Cemented fixation was also associated with lower odds of revision at 90 days (OR 0.307, 95% CI 0.167–0.561), 1 year (OR 0.430, 95% CI 0.261–0.707), and 3 years (OR 0.489, 95% CI 0.313–0.763). All-cause complications were significantly lower for cemented TKA at 90 days (OR 0.676, 95% CI 0.530–0.854), 1 year (OR 0.646, 95% CI 0.541–0.772), and 3 years (OR 0.736, 95% CI 0.630–0.860). No significant differences were observed between cohorts for aseptic loosening, periprosthetic fracture, or dislocation at any interval (Table 3).

**Table 3:** Outcome comparisons between cemented and uncemented TKA patients before matching

Outcome	Time	Cemented TKA, *n* (%)	Uncemented TKA, *n* (%)	OR (95% CI)
Dislocation	90 days	133 (0.11%)	≤ 10* (≈ 0.24%)	0.576 (0.303–1.096)
	1 year	409 (0.35%)	17 (0.41%)	0.952 (0.585–1.547)
	3 years	832 (0.71%)	37 (0.89%)	0.889 (0.639–1.237)
Prosthetic joint infection (PJI)	90 days	842 (0.72%)	51 (1.23%)	0.714 (0.537–0.948)
	1 year	1,574 (1.35%)	96 (2.32%)	0.644 (0.523–0.793)
	3 years	2,123 (1.82%)	114 (2.76%)	0.732 (0.605–0.886)
Aseptic loosening	90 days	122 (0.10%)	≤ 10* (≈ 0.24%)	0.560 (0.277–1.008)
	1 year	297 (0.25%)	18 (0.44%)	0.652 (0.405–1.050)
	3 years	676 (0.58%)	36 (0.87%)	0.742 (0.530–1.038)
Periprosthetic fracture	90 days	134 (0.11%)	≤ 10* (≈ 0.24%)	0.581 (0.305–1.104)
	1 year	271 (0.23%)	13 (0.31%)	0.824 (0.472–1.439)
	3 years	482 (0.41%)	21 (0.51%)	0.908 (0.586–1.406)
Revision	90 days	85 (0.07%)	12 (0.29%)	0.307 (0.167–0.561)
	1 year	185 (0.16%)	17 (0.41%)	0.430 (0.261–0.707)
	3 years	260 (0.22%)	21 (0.51%)	0.489 (0.313–0.763)
All-cause complications (ACC)	90 days	1,139 (0.97%)	73 (1.77%)	0.676 (0.530–0.854)
	1 year	2,193 (1.88%)	133 (3.22%)	0.646 (0.541–0.772)
	3 years	3,249 (2.78%)	173 (4.18%)	0.736 (0.630–0.860)

* In order to protect patient privacy within the TriNetX database outcomes with 10 or fewer patients are mathematically treated as 10.

After propensity score matching, there were statistical differences in many of the outcomes between cemented and uncemented TKA. Cemented TKA remained associated with lower rates of PJI at both 90 days (OR = 0.525, 95% CI 0.334–0.824) and one year (OR = 0.695, 95% CI 0.505–0.956). Additionally, although the difference was not statistically significant, the odds ratio still favored cemented fixation by 3 years (OR = 0.780, 95% CI 0.588–1.036). Cemented TKA also had fewer all-cause complications at 90 days (OR = 0.568, 95% CI 0.361–0.824), one year (OR = 0.671, 95% CI 0.511–0.882), and three years (OR = 0.765, 95% CI 0.697–0.963). As shown in Table 4, no statistical differences were observed between cemented and uncemented TKA for the other outcomes including aseptic loosening, periprosthetic fracture, dislocation, and revision — at any time interval. Interpretation of rare outcomes should consider that TriNetX masks cell counts ≤ 10, which may result in mild overestimation of event rates for infrequent complications.

**Table 4:** Outcome comparisons between cemented and uncemented TKA patients after matching

Outcome	Time	Cemented TKA, *n* (%)	Uncemented TKA, *n* (%)	OR (95% CI)
Dislocation	90 days	≤ 10*	≤ 10*	1 (0.416–2.405)
	1 year	15	17	0.882 (0.44–1.768)
	3 years	33	37	0.891 (0.557–1.427)
Prosthetic joint infection (PJI)	90 days	29	55	0.525 (0.334–0.824)
	1 year	65	93	0.695 (0.505–0.956)
	3 years	87	111	0.780 (0.588–1.036)
Aseptic loosening	90 days	≤ 10*	≤ 10*	1 (0.416–2.405)
	1 year	16	18	0.889 (0.453–1.744)
	3 years	30	36	0.832 (0.512–1.353)
Periprosthetic fracture	90 days	≤ 10*	≤ 10*	1 (0.416–2.405)
	1 year	≤ 10*	13	0.769 (0.337–1.755)
	3 years	16	21	0.761 (0.397–1.46)
Revision	90 days	≤ 10*	12	0.833 (0.36–1.93)
	1 year	≤ 10*	17	0.587 (0.269–1.284)
	3 years	≤ 10*	21	0.475 (0.224–1.01)
All-cause complications (ACC)	90 days	44	47	0.568 (0.361–0.824)
	1 year	88	130	0.671 (0.511–0.882)
	3 years	131	170	0.765 (0.697–0.963)

* In order to protect patient privacy within the TriNetX database outcomes with 10 or fewer patients are mathematically treated as 10.

## Discussion

This study found that, in the matched cohort, cemented TKA demonstrated significantly lower rates of prosthetic joint infection (PJI) at 90 days and 1 year compared with uncemented TKA, along with lower all-cause complication rates at 90 days, 1 year, and 3 years. These findings provide additional clarity to an area of ongoing debate and contribute to the growing but still inconsistent body of literature comparing outcomes between cemented and uncemented fixation techniques. They also highlight that, despite technological advancements in uncemented implant design, meaningful differences in early complications may still exist between the two approaches.

Prior research has reported variable results regarding complications between fixation methods. Prasad et al. found no significant differences in revision, infection, or functional outcomes in a meta-analysis of 755 TKAs, though the small sample size limited statistical power for detecting rare events [11]. Wilczyński et al. similarly reported minimal differences in a narrative review of fixation techniques [13]. In contrast, recent large-database studies have reported higher early complication risks with uncemented fixation. Chiou et al. analyzed over 300,000 TKAs and found higher rates of reoperation and incision and drainage procedures within the first postoperative year in uncemented TKA [14]. A 2023 national database study by Forlenza et al. further demonstrated that cementless TKA was associated with a significantly increased risk of early aseptic loosening requiring revision within 2 years (OR 2.34) [12]. The present study aligns with these newer findings, suggesting that—at the population level—uncemented fixation may still be associated with higher early complication risk despite recent improvements in implant technology.

All-cause complications also demonstrated important differences between fixation types. While the early success of many new designs of un-cemented TKA has been attributed to better primary stability of the components due to either porosity or 3D-printed surfaces [3, 15], implant-specific details are not available within TriNetX. Therefore, we could not evaluate their individual performance and cannot conclude if all modern designs will perform similarly as they have in past studies of contemporary designs. Also, as recently shown in institutional studies, there still exists an ongoing variation in clinical results. In 2025, Salmons et al. found an increased rate of infection in the cementless TKAs, with a five-year survival without infection at 98% for cementless versus 99% for cemented fixation (Hazard Ratio = 3.0) [22]. This finding again highlights how there may exist a continued disparity in infection risk in spite of advancements in design.

Aseptic loosening remains a key point of discussion among fixation strategies. While several studies have suggested increased loosening rates with uncemented implants, especially when the bone is compromised or the initial fixation is poor [16, 17], our study was unable to demonstrate any statistically significant increase in loosening in the uncemented group after matching. However, loosening is heavily dependent upon variables not included in the TriNetX database, such as implant design and geometry, surface finish, bone quality, and surgical technique. Observational database findings should therefore be interpreted with caution.

Infection prevention strategies also differ between fixation methods, and cement-related factors may contribute to observed differences. Antibiotic loaded bone cement (ALBC) may contribute to the lower rates of infection seen with cemented TKA, because the release of antibiotics from the cement provides local anti-microbial protection [2]. However, evidence regarding ALBC remains mixed. Some studies report benefit, while others—including Namba et al. and Nourie et al.—have demonstrated no significant reduction in PJI risk compared with plain cement [19, 20]. The present study could not determine if the use of antibiotic cement in the cemented group had any effect on the PJI rates, since it is not captured in the TriNetX database. Other factors, such as fixation stability or patient selection, may also play meaningful roles.

Several limitations must be considered when interpreting these findings. The TriNetX database does not contain any information regarding the design of the implants, the type of polyethylene used, the geometric shape of the tibial base plate, the fixation techniques, the methods of cementing, the peri-operative protocols, or the volume of surgeons that performed the surgeries. Therefore, it is likely that there is residual confounding in terms of implant- or surgeon-specific factors. It is also possible that there is selection bias in that younger or healthier patients tend to receive un-cemented implants, while cemented fixation is more commonly used in older patients with poorer bone quality. Although the matching process significantly reduced the baseline differences, nicotine dependence was close to statistical significance suggesting some minor residual imbalance. As with all database studies, there is the possibility of variability in coding, incomplete follow-up, and missing data in small cell counts which may affect the trends that are observed. Importantly, other critical outcomes such as osteolysis, radiographic alignment, component migration, patient-reported function, and long-term survival of the implants were not available.

Despite these limitations, this study provides valuable mid-term (three-year) outcome data from a large, diverse population and aligns with recent evidence suggesting that cemented fixation may continue to offer lower rates of early complications. Further prospective studies with longer follow-up, implant-specific data, and standardized perioperative protocols are needed to clarify the long-term comparative performance of cemented and uncemented TKA. Fixation choice should remain individualized based on bone quality, comorbidities, activity level, implant design, and surgeon experience.

## Conclusion

In this large propensity-matched analysis, cemented TKA was associated with lower rates of prosthetic joint infection and all-cause complications at early and mid-term follow-up compared with uncemented TKA. No significant differences were observed in aseptic loosening or periprosthetic fracture. These findings are consistent with several contemporary large-database studies but remain subject to the inherent limitations of observational research. Further long-term, implant-specific comparative studies are needed to determine whether advances in cementless technology will meaningfully reduce complication risks over time. Until then, fixation choice should continue to be guided by patient-specific characteristics and surgeon expertise.

## Data Availability

No datasets were generated or analysed during the current study.

## Appendix

**Appendix 1** Current Procedural Terminology (CPT) and ICD-10 (International Classification of Diseases, 10th Revision) codes.

**Table:**

Complication	CPT and ICD-10 codes
Dislocation	T84.02, 0.029, 0.029 A, .029D
Prosthetic Joint Infection (PJI)	T84.53, 0.54
Loosening	T84.032, 0.032 A, .032D, 0.032 S, 0.033, 0.033 A, .033D, 0.033 S
Periprosthetic Fracture	M97.1, M97.11, M97.12
Revision	0SWC4JC, 0SWV0JZ, 0SWCXJZ, 0SWC0JC, 0SWCXJC, 0SWT0JZ, 0SWTXJZ, 0SWC4JZ, 0SWC4JZ, 0SWCXJZ, 0SWC3JZ, 0SWC0JZ, 0SWD0JZ, 0SWD3JZ, 0SWDXJZ, 0SWD4JZ, 0SWUXJZ, 0SWU0JZ, 0SWDXJC, 0SWD0JC, 0SWWXJZ, 0SWW0JZ, 0SWD4JC
All Cause Complications	Sum of the other variables

.
